# Clinical features of chronic enteropathy associated with *SLCO2A1* gene: a new entity clinically distinct from Crohn’s disease

**DOI:** 10.1007/s00535-017-1426-y

**Published:** 2018-01-08

**Authors:** Junji Umeno, Motohiro Esaki, Atsushi Hirano, Yuta Fuyuno, Naoki Ohmiya, Shigeyoshi Yasukawa, Fumihito Hirai, Shuji Kochi, Koichi Kurahara, Shunichi Yanai, Keiichi Uchida, Shuhei Hosomi, Kenji Watanabe, Naoki Hosoe, Haruhiko Ogata, Tadakazu Hisamatsu, Manabu Nagayama, Hironori Yamamoto, Daiki Abukawa, Fumihiko Kakuta, Kei Onodera, Toshiyuki Matsui, Toshifumi Hibi, Tsuneyoshi Yao, Takanari Kitazono, Takayuki Matsumoto, The CEAS study group, Hiroyuki Kobayashi, Takashi Watanabe, Kunihiko Aoyagi, Hidehisa Ooi, Masano Akamatsu, Toshihiro Inokuchi, Sakiko Hiraoka, Hiroyuki Imaeda, Eiko Okimoto, Katsuya Endo, Tatsuki Mizuochi, Naohiko Harada, Tomoyuki Tsujikawa, Takeaki Ishii, Toshiyuki Matsui, Mitsuo Iida, Toshifumi Hibi

**Affiliations:** 1 Department of Medicine and Clinical Science, Graduate School of Medical Sciences, Kyushu University, Fukuoka, Japan; 2 Department of Gastroenterology, Fujita Health University School of Medicine, Toyoake, Japan; 3 Department of Gastroenterology, Fukuoka University Chikushi Hospital, Chikushino, Japan; 4 Department of Gastroenterology, Matsuyama Red Cross Hospital, Matsuyama, Japan; 5 Division of Gastroenterology, Department of Internal Medicine, School of Medicine, Iwate Medical University, Morioka, Japan; 6 Department of Gastrointestinal and Pediatric Surgery, Mie University Graduate School of Medicine, Tsu, Japan; 7 Department of Gastroenterology, Osaka City University Graduate School of Medicine, Osaka, Japan; 8 Department of Intestinal Inflammation Research, Hyogo College of Medicine, Nishinomiya, Japan; 9 Center for Diagnostic and Therapeutic Endoscopy, Keio University School of Medicine, Tokyo, Japan; 10 The Third Department of Internal Medicine, Kyorin University School of Medicine, Mitaka, Japan; 11 Division of Gastroenterology, Department of Medicine, Jichi Medical University, Tochigi, Japan; 12 Department of General Pediatrics, Miyagi Children’s Hospital, Sendai, Japan; 13 Department of Gastroenterology and Hepatology, Sapporo Medical University School of Medicine, Sapporo, Japan; 14 Center for Advanced IBD Research and Treatment, Kitasato University, Kitasato Institute Hospital, Tokyo, Japan; 15 Sada Hospital, Fukuoka, Japan

**Keywords:** Chronic nonspecific multiple ulcers of the small intestine, Crohn’s disease, Primary hypertrophic osteoarthropathy, Pachydermoperiostosis, Prostaglandin transporter

## Abstract

**Background:**

Chronic enteropathy associated with *SLCO2A1* gene (CEAS) is a hereditary disease caused by mutations in the *SLCO2A1* gene and characterized by multiple small intestinal ulcers of nonspecific histology. *SLCO2A1* is also a causal gene of primary hypertrophic osteoarthropathy (PHO). However, little is known about the clinical features of CEAS or PHO.

**Methods:**

Sixty-five Japanese patients recruited by a nationwide survey of CEAS during 2012–2016 were enrolled in this present study. We reviewed the clinical information of the genetically confirmed CEAS patients.

**Results:**

We identified recessive *SLCO2A1* mutations at 11 sites in 46 patients. Among the 46 patients genetically confirmed as CEAS, 13 were men and 33 were women. The median age at disease onset was 16.5 years, and parental consanguinity was present in 13 patients (28%). Anemia was present in 45 patients (98%), while a single patient experienced gross hematochezia. All patients showed relatively low inflammatory markers in blood tests (median CRP 0.20 mg/dl). The most frequently involved gastrointestinal site was the ileum (98%), although no patient had mucosal injuries in the terminal ileum. Mild digital clubbing or periostosis was found in 13 patients (28%), with five male patients fulfilling the major diagnostic criteria of PHO.

**Conclusions:**

The clinical features of CEAS are distinct from those of Crohn’s disease. Genetic analysis of the *SLCO2A1* gene is therefore recommended in patients clinically suspected of having CEAS.

**Electronic supplementary material:**

The online version of this article (10.1007/s00535-017-1426-y) contains supplementary material, which is available to authorized users.

## Introduction

Chronic enteropathy associated with *SLCO2A1* gene (CEAS) was initially described as “chronic nonspecific multiple ulcers of the small intestine” in 1968 [1]. Recently, it has become evident that the disease is caused by loss-of-function mutations in the *SLCO2A1* gene, which encodes a prostaglandin transporter [2]. CEAS is a rare, intractable disease characterized by multiple small intestinal ulcers of nonspecific histology and chronic persistent gastrointestinal (GI) bleeding [3, 4]. Its symptoms, including general fatigue, edema, and abdominal pain, typically appear during adolescence and the clinical course is chronic and intractable. To date, the diagnosis of CEAS has been based on clinical symptoms and confirmation of small bowel lesions compatible with the disease. Because CEAS mimics ileal Crohn’s disease (CD) with respect to ileal ulcers and stenosis [5, 6], it is often difficult to distinguish CEAS from CD by clinical features alone.

Since the identification of *SLCO2A1* mutation as a cause of CEAS, it has become possible to distinguish the disease from other enteropathies, including CD. The prostaglandin transporter coded by *SLCO2A1* mediates the efflux of newly synthesized prostaglandins from cells, epithelial prostaglandin transport, prostaglandin clearance, and prostaglandin degradation [7, 8]. Homozygous or compound heterozygous mutations in *SLCO2A1* are known to cause not only CEAS but also a subtype of primary hypertrophic osteoarthropathy (PHO) [9]. PHO, also known as pachydermoperiostosis, is an autosomal recessive inherited disease that affects the skin and bones, presenting digital clubbing, periostosis, acroosteolysis, painful joint enlargement, and thickened skin. We have previously reported that some patients with CEAS also have clinical features of PHO as extra-intestinal manifestations [2, 10]. While CEAS and PHO share a common causative gene, little is known about the clinical features of CEAS. We therefore conducted a nationwide survey in Japan to investigate the clinical manifestations of CEAS.

## Materials and methods

### Study participants and clinical data

During the period 2012–2016, we conducted a Japanese nationwide survey for CEAS at the initiative of the research group for rare and intractable diseases at the Japan Agency for Medical Research and Development (AMED). At a nationwide congress of gastroenterologists specializing in inflammatory bowel disease, we reached a common consensus for the diagnostic criteria of CEAS. During the subsequent period, we established a database of patients suspected of having CEAS and recruited 65 such patients from 31 institutions.

Blood samples from all participants were collected from participating institutions. Patients who were recruited for our previous investigations [2, 11, 12] were also included in this study. We screened these 65 patients from 62 unrelated families for *SLCO2A1* gene mutations. The diagnosis of CEAS was based on the published clinical criteria and genetic analysis (Supplementary Table S1) [2, 3, 13]. Clinical data including age at diagnosis and at disease onset, presence of consanguinity and family history, nonsteroidal anti-inflammatory drugs (NSAIDs) use, history of *Helicobacter pylori* (*H*. *pylori*) infection, symptoms, laboratory data at diagnosis, and surgical history were collected.

Information concerning GI involvement, as determined by radiographic or endoscopic examinations, was also collected. GI involvement of CEAS was considered positive if any active ulcerative lesion or obvious scarred ulcer was observed. The site of small intestinal involvement was determined as the terminal ileum when small bowel lesions were observed by conventional ileocolonoscopy.

We also determined whether patients had clinical manifestations of PHO, such as digital clubbing, periostosis, acroosteolysis, arthralgia of large joints, knee-joint effusions, hyperhidrosis, pachydermia, seborrhea, acne, flushing, and history of patent ductus arteriosus and delayed cranial suture closure. Periostosis and acroosteolysis were assessed by X-ray evaluation.

All study participants provided written informed consent for genetic analysis. The study protocol was approved by the ethics committee of each participating institution.

### *SLCO2A1* gene mutation analysis

DNA was extracted from peripheral blood using standard methods. Thirteen pairs of primers were designed using Primer3web (http://primer3.ut.ee/) to amplify all 14 coding exons and intron–exon boundaries of the *SLCO2A1* gene (Supplementary Table S2). Each PCR reaction mixture included 10 ng of genomic DNA, 10 pmol of each primer, 10 µl of Gflex PCR buffer (Mg^2+^, dNTP plus), and 0.5 unit of Tks Gflex DNA polymerase (Takara, Shiga, Japan) in a final volume of 20 µl. The PCR reaction mixture was initially incubated at 94 °C for 1 min, followed by 35 cycles of denaturation at 98 °C for 10 s, and both annealing and extension at 68 °C for 30 s. PCR products were purified with an Exo-SAP Cleanup kit (Affymetrix, Cleveland, OH, USA) and sequenced using a BigDye Terminator v3.1 cycle sequencing kit and an ABI 3730xl DNA analyzer (Applied Biosystems, Foster City, CA, USA). Sequence analysis was performed using DNA Baser v4.36 software (Heracle BioSoft, Mioveni, Arges, Romania). We focused primarily on non-synonymous and splice-site variants that may alter protein function. The called variants with allele frequency of more than 1% in dbSNP147 (http://www.ncbi.nlm.nih.gov/projects/SNP/) were excluded. The effect of each missense mutation was predicted using SIFT (http://sift.jcvi.org/) [14], PolyPhen-2 (http://genetics.bwh.harvard.edu/pph2/) [15], and PROVEAN (http://provean.jcvi.org/) [16] software tools.

### Statistics

To explore the association between types of *SLCO2A1* gene mutations and clinical phenotypes, patients were divided into two groups by homozygous c.940 + 1G > A mutation. Fisher’s exact test and the Mann–Whitney *U* test were used to analyze categorical data and quantitative data between the two groups, respectively. The analyses were performed using the JMP Pro statistical package 12.2.0 (SAS Institute, Cary, NC, USA). Values of *p* < 0.05 were regarded as statistically significant.

## Results

### *SLCO2A1* gene mutation analysis

Among 65 patients with suspected CEAS, we identified 46 patients from 43 unrelated families with homozygous or compound heterozygous *SLCO2A1* mutations. The identified *SLCO2A1* mutations were located at 11 sites (Table 1). The minor allele frequencies of these mutations were absent or were less than 0.01% in the dbSNP147 database. Among the identified *SLCO2A1* mutations, two splice site mutations (c.940 + 1G > A and c.1461 + 1G > C), two frameshift mutations (c.830dupT and c.830delT), and three nonsense mutations (c.421G > T, c.770G > A and c.1807C > T) were predicted to result in a stop codon and to produce truncated proteins (Supplementary Figure S1). The remaining four missense mutations were predicted to be deleterious according to SIFT, PolyPhen-2, and PROVEAN. Thus, all identified mutations were considered to cause loss of function. We therefore diagnosed these 46 patients as having CEAS. Two of the 11 identified mutations, c.97G > C and c.770G > A, were novel *SLCO2A1* gene mutations. Twenty-four patients (52%) had homozygous mutations and the remaining 22 patients (48%) had compound heterozygous mutations. The most frequent mutation was c.940 + 1G > A (50/92 = 54%), and 17 patients (37%) had this mutation in the homozygous form.

**Table 1:** Identified *SLCO2A1* gene mutations in 46 patients with CEAS

No.	Genomic position chr3 (hg19)	Site	Nucleotide change	Predicted effect		Mutant allele frequency	dbSNP	Mutant allele frequency^b^
1	133,698,462	Exon 2	c.97G > C	p.V33L	Deleterious^a^	1/92	–	0
2	133,674,014	Exon 4	c.421G > T	p.E141X	Truncated	2/92	–	1/2198 (0.045%)
3	133,673,888	Exon 4	c.547G > A	p.G183R	Deleterious^a^	1/92	–	0
4	133,672,567	Exon 5	c.664G > A	p.G222R	Deleterious^a^	6/92	–	1/2192 (0.046%)
5	133,670,143	Exon 6	c.770G > A	p.W257X	Truncated	1/92	–	0
6	133,670,083	Exon 7	c.830dupT	p.F277Lfs^a^17	Truncated	6/92	rs751192029	1/2280 (0.044%)
7	133,670,083	Exon 7	c.830delT	p.F277Sfs^a^6	Truncated	1/92	rs765906270	0
8	133,667,736	Intron 7	c.940 + 1G > A	Splice site	Truncated	50/92	rs765249238	2/2188 (0.091%)
9	133,664,028	Exon 10	c.1372G > T	p.V458F	Deleterious^a^	2/92	–	0
10	133,663,938	Intron 10	c.1461 + 1G > C	Splice site	Truncated	2/92	–	0
11	133,654,625	Exon 13	c.1807C > T	p.R603X	Truncated	20/92	rs776813259	0

^a^Mutation pathogenicity according to SIFT, PolyPhen-2, and PROVEAN

^b^Data from the Human Genetic Variation Database (HGVD) for the Japanese population (version 2.1)

### Clinical features

Of the 46 patients genetically confirmed as CEAS, 13 were men and 33 were women, a male–female ratio of 1:2.5 (Table 2). The median age at disease onset was 16.5 years (range, 1–69 years) and parental consanguinity was present in 13 patients (28%). Although almost all patients presented with anemia; hematochezia was observed in one patient. According to the available clinical information, no patients received any NSAIDs at the time of diagnosis. Five of 20 patients who checked *H*. *pylori* status were positive for *H*. *pylori* infection. The median hemoglobin and serum protein levels at diagnosis were 9.6 and 5.2 g/dl, respectively. The median CRP level was 0.20 mg/dl (range, 0–1.6 mg/dl). Among the 46 patients with CEAS, 29 (63%) had undergone one or more small bowel surgeries.

**Table 2:** Clinical findings of CEAS patients (*n* = 46)

Sex male/female	13/33
Age at diagnosis (years, median)	40 (7–69)
Age at onset (years, median)	16.5 (1–69)
Consanguinity	13 (28%)
Family history	10 (22%)
Past history of NSAIDs use^b^	2 (4.5%)
NSAIDs use at diagnosis^c^	0 (0%)
History of *H*. *pylori* infection^d^	5 (24%)
Symptoms	
Anemia	45 (98%)
Abdominal pain	18 (39%)
Edema	11 (24%)
Diarrhea	2 (4%)
Hematemesis	1 (2%)
Hematochezia	1 (2%)
Laboratory data at diagnosis	
Hemoglobin (g/dl, median)	9.6 (2.3–13.7)
Serum protein (g/dl, median)	5.2 (2.7–8.2)
CRP (mg/dl, median)	0.20 (0–1.6)
Surgery	29 (63%)
Extra-intestinal manifestations	
Digital clubbing^a^	10 (22%)
Periostosis^a,e^	11 (25%)
Acroosteolysis^e^	1 (2%)
Arthralgia of large joints	7 (15%)
Knee-joint effusions	4 (9%)
Hyperhidrosis	4 (9%)
Pachydermia^a^	8 (17%)
Seborrhea	3 (7%)
Acne	7 (15%)
Flushing	4 (9%)
Patent ductus arteriosus	1 (2%)
Delayed cranial suture closure	0

^a^These manifestations are included in the major clinical criteria for PHO

Data are available for ^b^ 44, ^c^ 45, and ^d^ 21 patients, respectively

^e^Data are available for 44 patients with X-ray evaluation

**Fig. 1:**
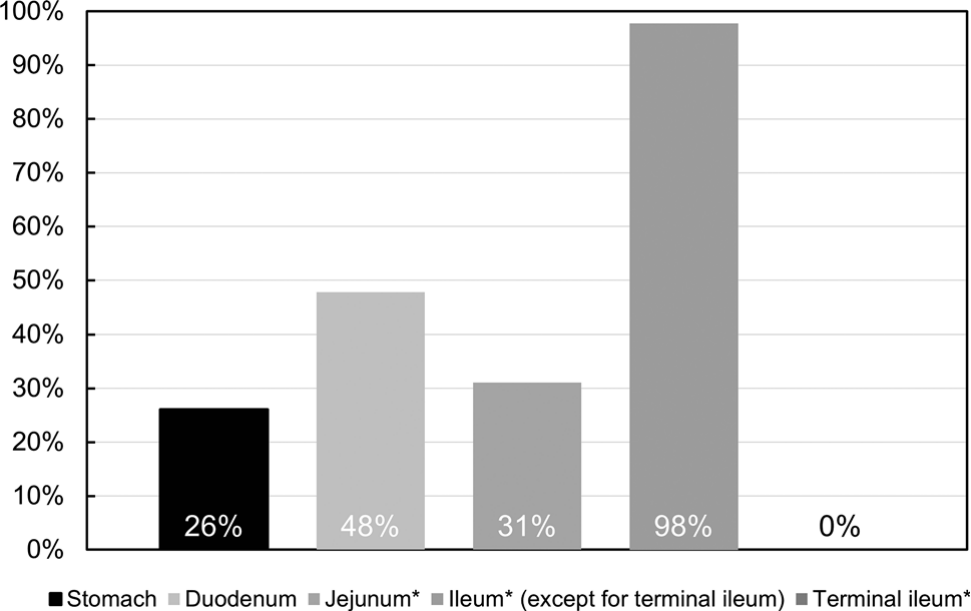
Involved sites in the gastrointestinal tract in CEAS (*n* = 46). *Data are available for 45 patients

Figure 1 indicates the frequency of disease involvement in each site of the GI tract. Although the small intestine was involved in all patients and the ileum was most frequently involved (98%), the terminal ileum was not involved. Gastric involvement was significantly more frequent in patients positive for *H*. *pylori* infection compared to patients negative for *H*. *pylori* infection (60 vs. 6.3%, *p* = 0.028). Conversely, duodenal involvement was less frequently observed in patients with *H*. *pylori* infection (20 vs. 6%, not significant). No active ulceration was found in the esophagus or in the large bowel.

**Fig. 2:**
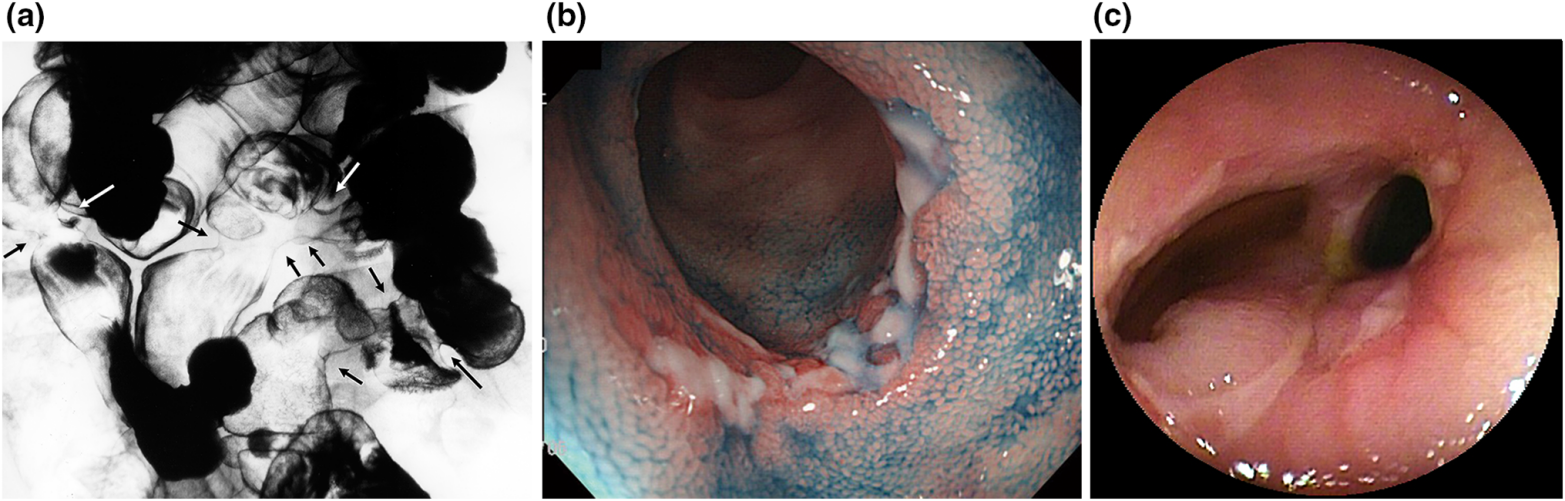
Radiographic and endoscopic findings of small intestinal lesions. **a** Double-contrast radiography depicts multiple deformities and strictures at the distal jejunum and ileum (*arrows*). **b**, **c** Endoscopic findings. Shallow circular ulcers (**b**) or circular and oblique ulcers with symmetrical deformity and pseudodiverticulum formation (**c**) in patients with CEAS. Figures are reprinted with permission from Refs. [10]

Figure 2 shows the typical radiographic and endoscopic findings of CEAS. Under radiographic examination, small intestinal lesions were typically observed as multiple deformities or stenoses, located at the distal jejunum to the ileum (Fig. 2a). The lesions occurred asymmetrically and independently of the mesenteric side and were endoscopically recognized as shallow ulcers with or without luminal narrowing, as reported previously [6, 12]. Ulcerative lesions varied in shape, being circular, oblique, or longitudinal, and ulcers occasionally formed a pseudodiverticulum (Fig. 2b, c).

**Fig. 3:**
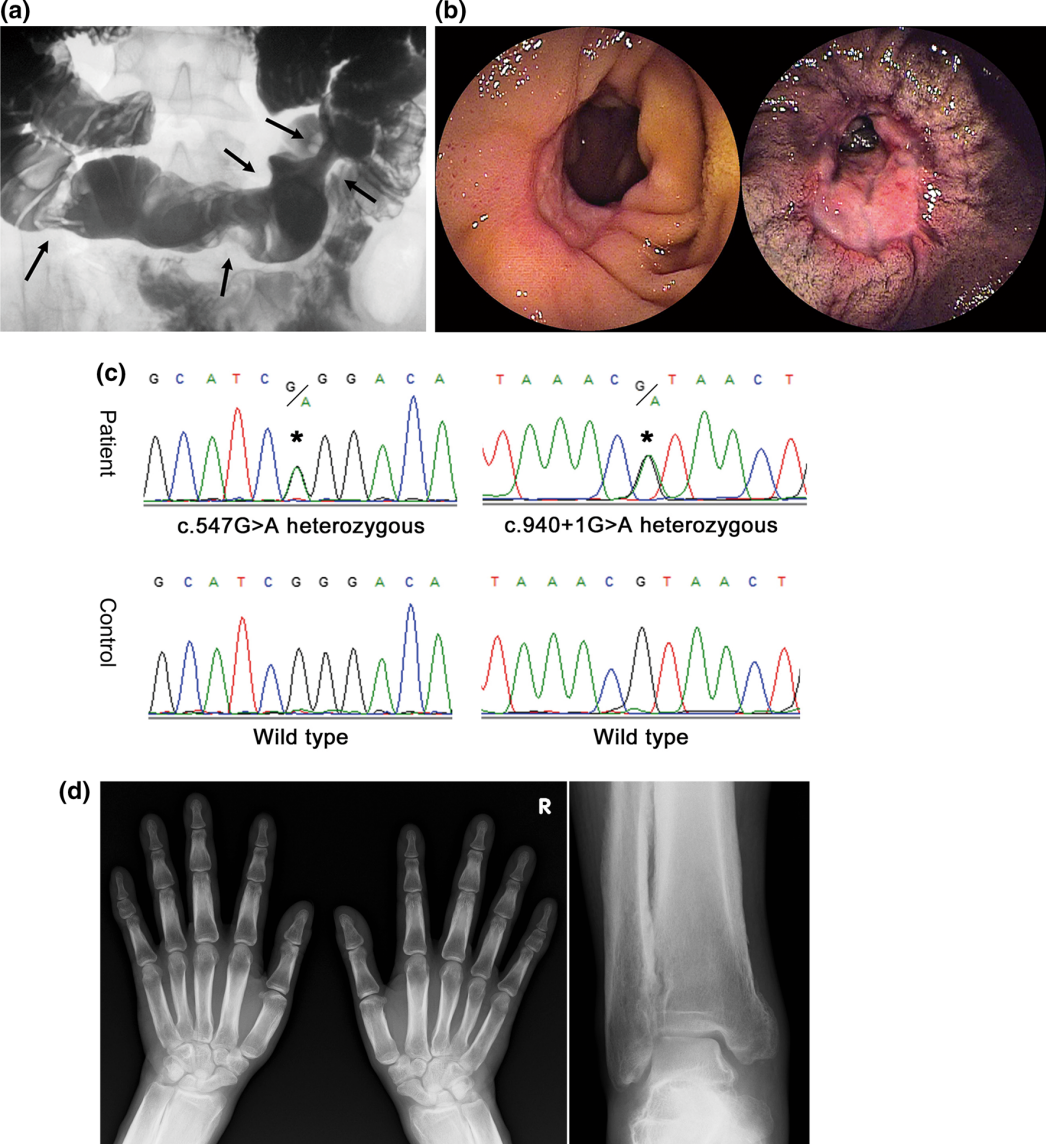
Clinical findings of a male patient (26 years) with CEAS and PHO. **a** Images of small bowel follow through. Filling image showing multiple eccentric rigidities in the ileum (*arrows*). **b** Images of double-balloon enteroscopy. Two lesions of ulceration with stenosis were observed in the ileum. **c** Sanger sequencing of the *SLCO2A1* gene. This patient had compound heterozygous mutations, namely c.547G > A (p.G183R) and c.940 + 1G > A (splice site). **d** X-ray images of the extremities. Radiographs of both the hands and ankle joints revealed cortical hyperostosis and periosteal reaction. Figures are reprinted with permission from Ref. [17] and [18]

Any one or more of the clinical manifestations of digital clubbing, periostosis, and pachydermia were present in 14 (30%) of 44 patients, who underwent X-ray examination (Table 2). Five male patients (11%) had all three clinical manifestations, thus fulfilling the major clinical criteria of PHO (Fig. 3). On the other hand, no female patient fulfilled the major criteria of PHO. No patient required treatment for these extra-intestinal manifestations.

To evaluate the influence of disease onset on clinical features, we divided the patients into an early onset group (age at onset < 20; 27 patients) and a late-onset group (age at onset ≥ 20 years; 19 patients), and compared clinical features between the groups. The surgical rate was significantly higher in the early onset group than in the late-onset group (78 vs. 42%, *p* = 0.028, Supplementary Table S3), however, no other clinical features were different between the two groups.

### Sex differences in clinical features

Table 3 shows the gender differences in clinical features of CEAS. Gastric involvement was significantly lower in males than in females (0 vs. 36%, *p* = 0.01). Serum protein level was significantly lower in females than in males (median 5.0 vs. 5.5 g/dl, *p* = 0.009). In contrast, major manifestations of PHO were more frequently found in males than in females (digital clubbing 54 vs. 9%, *p* = 0.003; periostosis 54 vs. 13%, *p* = 0.008; pachydermia 62 vs. 0%, *p* < 0.0001). There was no difference in age at diagnosis and age at disease onset between males and females.

**Table 3:** Comparison of clinical findings of CEAS patients by sex

	Male (*n* = 13)	Female (*n* = 33)	p*
Age at diagnosis (years, median)	31	40.5	NS
Age at onset (years, median)	14	19	NS
Consanguinity	3 (23%)	10 (30%)	NS
Family history	3 (23%)	7 (21%)	NS
Past history of NSAIDs use^†^	1/13 (7.7%)	1/31 (3.2%)	NS
History of *H*. *pylori* infection^‡^	1/11 (9.1%)	4/10 (40%)	NS
Symptoms			
Abdominal pain	4 (31%)	14 (42%)	NS
Disease site			
Stomach	0	12 (36%)	**0.01**
Duodenum	8 (62%)	14 (42%)	NS
Jejunum^§^	5 (42%)	9 (27%)	NS
Ileum^§^ (except for terminal ileum)	11 (92%)	33 (100%)	NS
Laboratory data at diagnosis			
Hemoglobin (g/dl, median)	10.2 (2.3–13.5)	9.5 (4.8–13.7)	NS
Serum protein (g/dl, median)	5.5 (4.8–8.2)	5.0 (2.7–6.7)	**0.009**
CRP (g/dl, median)	0.26 (0–1.6)	0.20 (0–1.1)	NS
Surgery	6 (46%)	23 (70%)	NS
c.940 + 1G > A homozygous mutation	7 (54%)	10 (30%)	NS
Extra-intestinal manifestations			
Digital clubbing	7 (54%)	3 (9.1%)	**0.003**
Periostosis|	7 (54%)	4 (13%)	**0.008**
Arthralgia of large joints	2 (15%)	5 (15%)	NS
Pachydermia	8 (62%)	0	**<** **0.0001**

*NS* not significant

Significant *p* values are indicated in bold

* Fisher’s exact test or Mann–Whitney *U* test

Data are available for ^†^44, ^‡^21 and ^§^45 patients, respectively

|Data are available for 44 patients with X-ray evaluation

### Association between *SLCO2A1* gene mutation and clinical features

Since c.940 + 1G > A was the most frequent mutation in the present study, we compared clinical features between two groups: one comprising patients with homozygous c.940 + 1G > A mutation and the other comprising those without. Serum protein level at diagnosis was significantly higher in the group with c.940 + 1G > A homozygotes than in the group without (median 5.4 vs. 5.1 g/dl, *p* = 0.03, Table 4). No other clinical features were different between the two groups.

**Table 4:** Comparison of clinical findings of CEAS patients by c.940 + 1G > A mutation

	c.940 + 1G > A homozygotes group (*n* = 17)	Non-c.940 + 1G > A homozygotes group (*n* = 29)	p*
Age at diagnosis (years, median)	38	42.5	NS
Age at onset (years, median)	14	19	NS
Symptoms			
Abdominal pain	8 (47%)	10 (34%)	NS
Disease site			
Stomach	4 (24%)	8 (28%)	NS
Duodenum	11 (65%)	11 (38%)	NS
Jejunum^†^	3 (18%)	11 (39%)	NS
Ileum^†^ (except for terminal ileum)	16 (94%)	28 (100%)	NS
Laboratory data at diagnosis			
Hemoglobin (g/dl, median)	10.7 (4.8–13.5)	9.4 (2.3–13.7)	NS
Serum protein (g/dl, median)	5.4 (1.2–8.2)	5.0 (2.7–7.0)	**0.03**
CRP (g/dl, median)	0.20 (0–1.6)	0.20 (0–1.1)	NS
Surgery	11 (65%)	18 (62%)	NS
Extra-intestinal manifestations			
Digital clubbing	3 (18%)	7 (24%)	NS
Periostosis	4 (24%)	7 (26%)	NS
Arthralgia of large joints	4 (24%)	3 (10%)	NS
Pachydermia	4 (24%)	4 (14%)	NS

*NS* not significant

A significant *p* value is indicated in bold

* Fisher’s exact test or Mann–Whitney *U* test

^†^Data are available for 45 patients

## Discussion

CEAS is a rare disease entity characterized by multiple intractable small intestinal ulcers caused by *SLCO2A1* gene mutations [2]. This disorder has been previously referred to as “chronic nonspecific multiple ulcers of the small intestine” because of the lack of specific histological findings such as granuloma and eosinophilic infiltration [1, 6]. Furthermore, the rarity of the disease and the term “nonspecific” in its nomenclature has elicited misunderstanding in the interpretation of the disease as various other conditions with multiple small intestinal ulcers of obscure origin [4]. Because CEAS is genetically distinct from other GI disorders, precise recognition of its clinical features and GI pathologies appears mandatory for a correct diagnosis.

In this study, we identified 11 different *SLCO2A1* gene mutations in 46 patients with CEAS from 43 families. As in previous reports [1, 3], we confirmed that CEAS occurs predominantly in females, and most patients manifest anemia without gross hematochezia. It was also evident that the disease is characterized by minimal inflammatory reactions. CEAS has been reported to most commonly develop in adolescence, and in fact the median age at disease onset was 16.5 years in the present study. However, the age at disease onset varied widely from 1 to 69 years. Furthermore, the present study demonstrated a lower rate of parental consanguinity among patients with CEAS than that reported previously [19]. Considering such ambiguous clinical features of the disease, it seems likely that CEAS is a GI disorder that should be distinguished from other enteropathies, despite being a hereditary disease.

Widespread use of capsule endoscopy and balloon-assisted enteroscopy has enabled the precise observation of small intestinal mucosal injuries. Small intestinal mucosal injuries can occur in various GI disorders, including CD, intestinal tuberculosis, vasculitis, NSAID-induced enteropathy, and CEAS. Distinction between ileal CD and CEAS seems indispensable, as neither anti-tumor necrosis factor-α antibody therapy nor immunomodulators are effective against CEAS. Small intestinal ulcers of CEAS have been typically described as circular or obliquely shaped shallow ulcers with clear margins which occur asymmetrically, regardless of mesenteric or anti-mesenteric side [3, 4, 12]. In contrast, small intestinal ulcers in CD are typically longitudinal and commonly found on the mesenteric side [20]. However, a clear distinction between CD and CEAS based solely on the morphologic features of the small intestinal lesions appears to be difficult. In the present study, we reconfirmed the sparing of the terminal ileum in CEAS while multiple ulcers occurred in the ileum [1, 3]. Since the terminal ileum is the most frequently involved site in CD, such an obvious difference in the site of ileal involvement may represent a strategy to distinguish CEAS from CD.

PHO is an autosomal recessive inherited disease that is classified into two subtypes based on its causal gene, one being the *HPGD* gene and the other the *SLCO2A1* gene [9, 21]. Because 15-hydroxyprostaglandin dehydrogenase encoded by *HPGD* is the main enzyme for prostaglandin degradation, systemic prostaglandin E2 levels are increased in patients with mutations in *HPGD* [21]. Similarly, mutations in *SLCO2A1* can increase systemic prostaglandin E2 levels by disturbing prostaglandin metabolism via a deficient transmembrane prostaglandin transporter. Clinical features of PHO including digital clubbing, periostosis, and pachydermia are likely to be the result of persistently elevated serum prostaglandin E2 levels. Moreover, male patients generally show more severe manifestations because prostaglandin E2 production is greater in males than in females [21]. As opposed to the clinical manifestations of PHO, we identified female predominance in the gastric involvement of CEAS in the present study. Considering that CEAS and PHO are autosomal recessive inherited disorders with shared causal gene mutations, the possible influence of sex-related modifier genes or hormones should be seriously considered for differences in major clinical manifestations according to gender. Further analyses for CEAS and PHO are warranted to clarify phenotypic differences according to gender.

Among *SLCO2A1* mutations, a splice-site mutation at intron 7 (c.940 + 1G > A; rs765249238) was the most frequently observed among CEAS patients, with 54% of mutant allele frequency. This mutation induces the deletion of the entire exon 7 of *SLCO2A1*, leading to a frameshift at amino acid position 288 and the introduction of a premature stop codon after six amino acid residues (p.R288Gfs*7) [2]. Based on data from the HGVD database [22], the allele frequency of c.940 + 1G > A in the Japanese population is 0.091% (2/2188). As this mutation is not observed in European or American populations [23], the prevalence rate of CEAS in the Japanese population might be higher than that in Caucasian populations. However, a case of an ethnically ambiguous family containing three male patients with clinical features of both CD and PHO has been reported [24]. Although it remains uncertain whether these patients had *SLCO2A1* mutations, the family members had actually been suffering from CEAS.

There are several limitations in the present study. First, the morphologic features of GI lesions could not be evaluated in detail because a full set of endoscopic or radiographic images was not obtained in this nationwide survey. We thus focused on reconfirming the distribution of GI involvement in the present study. Second, the status of *H*. *pylori* infection, which presumably affects the incidence of upper GI involvement, could not be identified in all patients. To clarify the true incidence and gender difference of GI involvement in CEAS, further studies with patients negative for *H*. *pylori* infection are required. Third, extra-intestinal manifestations found as clinical features of PHO were not evaluated in a formatted manner, suggesting a certain misclassification bias. However, at least one of the three major criteria of PHO was assessed by X-ray evaluation. We thus believe that the presence of those features was appropriately determined in the present study. Finally, the present study included a relatively small number of patients. While we understand that this is the largest case series of CEAS reported to date, further surveillance including international collaboration is necessary.

In conclusion, this nationwide survey verified the clinical features of CEAS using genetically confirmed patients. We also reconfirmed the female predominance in GI involvement in the disease. Although CEAS is a rare hereditary disease, it should be considered when encountering patients with multiple small intestinal ulcers outside of the terminal ileum. In addition, genetic analysis of *SLCO2A1* is key to confirming the disease diagnosis.

## Electronic supplementary material

Below is the link to the electronic supplementary material.

Supplementary Figure S1. Schematic diagram of the *SLCO2A1* gene and identified mutations in CEAS patients. The sites of mutations are denoted by arrows, excluding two splice-site mutations. This figure was generated using Protter (http://wlab.ethz.ch/protter/start/). (TIFF 1782 kb)

Supplementary material 2 (DOCX 16 kb)
