# Supplementary figures and images for: Clinical features of chronic enteropathy associated with SLCO2A1 gene: a new entity clinically distinct from Crohn’s disease
Source: J Gastroenterol. 2018 Jan 8;53(8):907–15. doi: 10.1007/s00535-017-1426-y (PMC6061663; doi:10.1007/s00535-017-1426-y)

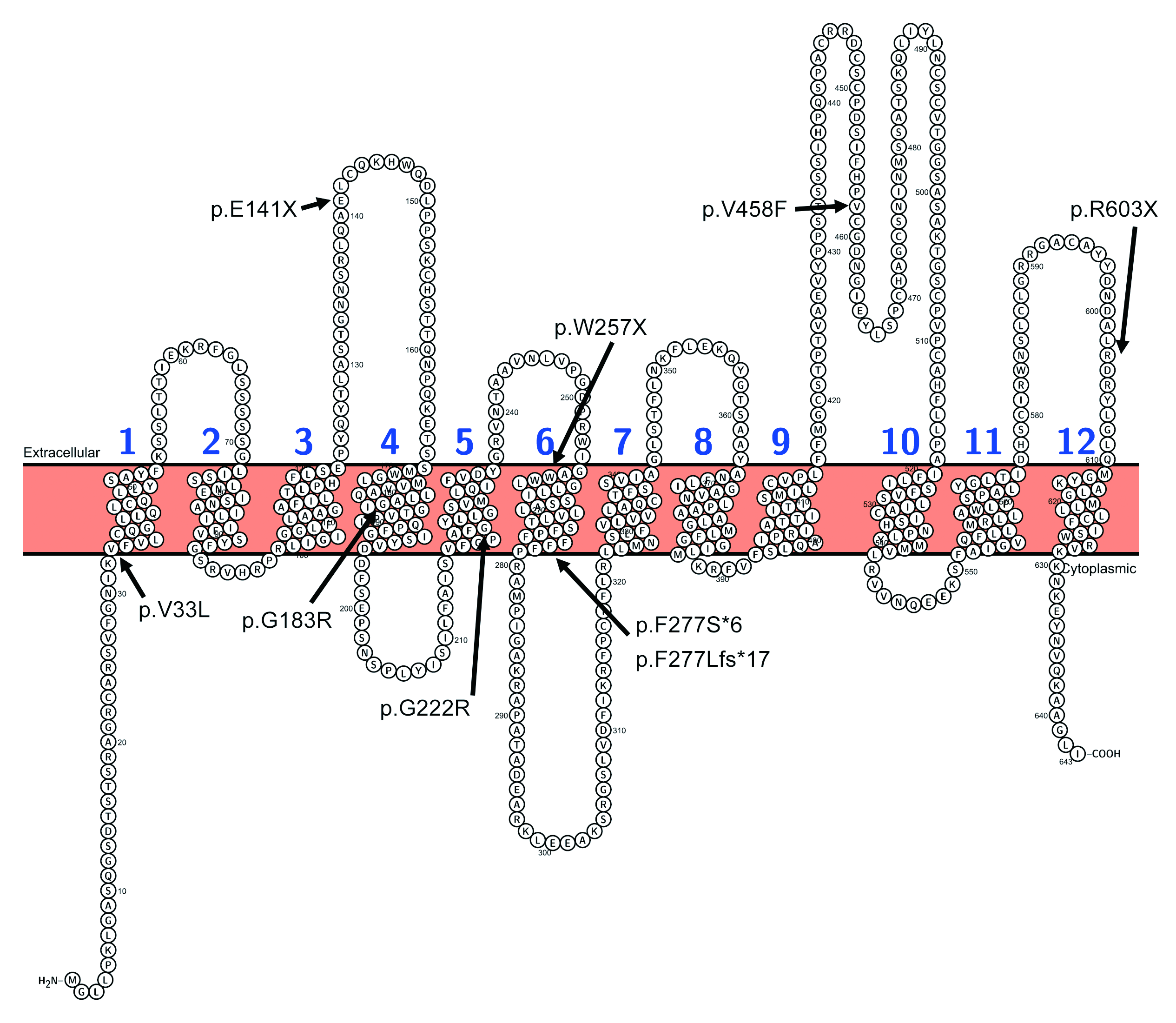

Supplement: Supplementary file 1 — Supplementary Figure S1. Schematic diagram of the SLCO2A1 gene and identified mutations in CEAS patients. The sites of mutations are denoted by arrows, excluding two splice-site mutations. This figure was generated using Protter (http://wlab.ethz.ch/protter/start/). (TIFF 1782 kb) [file 535_2017_1426_MOESM1_ESM.tif]
