# Supplementary material for: Clinical features of chronic enteropathy associated with SLCO2A1 gene: a new entity clinically distinct from Crohn’s disease
Source: J Gastroenterol. 2018 Jan 8;53(8):907–15. doi: 10.1007/s00535-017-1426-y (PMC6061663; doi:10.1007/s00535-017-1426-y)
Supplement: Supplementary file 2 — Supplementary material 2 (DOCX 16 kb) [file 535_2017_1426_MOESM2_ESM.docx]

Supplementary Table S1. Clinical criteria for chronic enteropathy associated with the *SLCO2A1* gene (CEAS)

| 1 | Persistent and occult blood loss from the GI tract except during bowel rest or postoperative period. | |
| --- | --- | --- |
| 2 | Confirmation of characteristic small intestinal lesions by macroscopy, radiography, or enteroscopy. | |
|  | i | Circular or oblique in alignment. |
|  | ii | Sharply demarcated from surrounding normal mucosa. |
|  | iii | Geographic or linear in shape. |
|  | iv | Multiplicity in number with < 4cm distance from each other. |
|  | v | Ulcers not reaching proper muscular layer. |
|  | vi | Scarred ulcers presumed to be the healing stage of those characterized by i–v* in cases treated by bowel rest. |
| 3 | Homozygous or compound heterozygous *SLCO2A1* mutations which are predicted to be deleterious. | |

*Depicted as symmetric and eccentric rigidity under small-bowel radiography, and concentric or nonconcentric stricture under enteroscopy. GI, gastrointestinal.

Supplementary Table S2. Primers for mutation analysis of the *SLCO2A1* gene

| Amplicon | Forward (5′–3′) | Reverse (5′–3′) | Size (bp) |
| --- | --- | --- | --- |
| Exon 1 | GCCTGGAGTTGTCCGAGTAAG | TTTGACACCCGAGGAAAAGAG | 620 |
| Exon 2 | ACAGAATTTGAAGGCGGACAG | TGATGACGTTTCATCCTGGAG | 655 |
| Exon 3 | AATGCTTTGATCCTCCTCCTG | CAGAGGGAAAACCAAAATTGC | 676 |
| Exon 4 | GCTGTTGGTTTAGGGTCCATC | CCCACATTCCACCTCTCTTTC | 786 |
| Exon 5 | AAGGGCAGAAAGTGAGTTTGC | GCAGGTCTCTTTGGAAGTTGG | 570 |
| Exon 6 | AATTTCAGCCACCCTCAACAC | CAATAGCTGGGAGAGGGAATG | 742 |
| Exon 7-8 | GATTGCCAACCAGGAAAACTG | AGGTGCTGTTTGCTTCAGGAG | 778 |
| Exon 9 | AGTTGCCACGTGAGAGATCAG | GTGTAGGCAAGGCAATCCTG | 860 |
| Exon 10 | TGCTTTGACTGGTTTCTGCTC | TGGTTGTCTTGACGCCTACTG | 747 |
| Exon 11 | GCAAAAGAACCTTGCACATTG | GCCTCCCTCTGCAATAAACAC | 710 |
| Exon 12 | AAACCGTCCACATGGATTTTG | CAGCATCCTTCTCTCCACTCC | 643 |
| Exon 13 | GTGGCCCTTCATGTTCTCTTC | GCCCGTGTATCTCCACTCTG | 564 |
| Exon 14 | GAAGGCAAATGAGGACTGGGG | ACCTGCTGCCGTTGTCATATG | 713 |

Supplementary Table S3. Comparison of clinical findings of CEAS patients by onset age

|  | early-onset group (n=27) | late-onset group (n=19) | *p** |
| --- | --- | --- | --- |
| Sex (Male / Female) | 9 / 18 | 4 / 15 | NS |
| Consanguinity | 7 (26%) | 6 (32%) | NS |
| Family history | 7 (26%) | 3 (16%) | NS |
| Symptoms |  |  |  |
| Abdominal pain | 11 (41%) | 7 (37%) | NS |
| Disease site |  |  |  |
| Stomach | 5 (19%) | 7 (37%) | NS |
| Duodenum | 15 (56%) | 7 (37%) | NS |
| Jejunum† | 9 (35%) | 5 (26%) | NS |
| Ileum (except for terminal ileum)† | 25 (96%) | 19 (100%) | NS |
| Laboratory data at diagnosis |  |  |  |
| Hemoglobin (g/dl, median) | 9.6 | 9.5 | NS |
| Serum protein (g/dl, median) | 5.1 | 5.2 | NS |
| CRP (g/dl, median) | 0.16 | 0.30 | NS |
| Surgery | 21 (78%) | 8 (42%) | **0.028** |
| c.940+1G>A homozygous mutation | 11 (41%) | 6 (32%) | NS |
| Extraintestinal manifestations |  |  |  |
| Digital clubbing | 5 (19%) | 5 (26%) | NS |
| Periostosis‡ | 5 (19%) | 6 (33%) | NS |
| Arthralgia of large joints | 4 (15%) | 3 (16%) | NS |
| Pachydermia | 6 (22%) | 2 (11%) | NS |

NS, not significant. *Fisher’s exact test or Mann-Whitney U test. Data are available for †45, and ‡44 patients, respectively. A significant *p* value is indicated in bold.
